# Iron Parameters in Patients Treated with Roxadustat for Anemia of Chronic Kidney Disease

**DOI:** 10.3390/jcm12134217

**Published:** 2023-06-22

**Authors:** Tomas Ganz, Francesco Locatelli, Mustafa Arici, Tadao Akizawa, Michael Reusch

**Affiliations:** 1 Department of Medicine, University of California Los Angeles, Los Angeles, CA 90095, USA; 2 Department of Nephrology and Dialysis, Alessandro Manzoni Hospital, 23900 Lecco, Italy; 3 Department of Nephrology, Hacettepe University, 06560 Ankara, Turkey; 4 Division of Nephrology, Department of Medicine, Showa University School of Medicine, Tokyo 142-8666, Japan; 5 Guard Therapeutics International AB, 114 39 Stockholm, Sweden

**Keywords:** anemia, chronic kidney disease, erythropoiesis-stimulating agent, iron, roxadustat

## Abstract

Roxadustat is a novel agent with a distinct mechanism of action compared to erythropoiesis-stimulating agents (ESAs) and a potentially different combination of effects on iron parameters. This narrative review describes the effects of roxadustat on iron parameters and on hemoglobin levels in the context of iron supplementation in patients with anemia of non-dialysis-dependent (NDD) or dialysis-dependent (DD) chronic kidney disease (CKD). Roxadustat use was associated with a greater reduction in serum ferritin levels than seen with ESAs and an increase in serum iron levels compared to a decrease with ESAs. Decreases in transferrin saturation in patients treated with roxadustat were relatively small and, in the case of patients with NDD CKD, not observed by Week 52. These changes reflect the concomitant increases in both serum iron and total iron-binding capacity. Compared to placebo and an ESA, roxadustat improved iron availability and increased erythropoiesis while requiring less intravenous iron use. Hepcidin levels generally decreased in patients who received roxadustat compared to baseline values in all CKD populations; these decreases appear to be more robust with roxadustat than with an ESA or placebo. The mechanisms behind the effects of roxadustat and ESAs on iron availability and stores and erythropoiesis appear to differ and should be considered holistically when treating anemia of CKD.

## 1. Introduction

### 1.1. Roxadustat and Its Use in Clinical Studies in Anemia of Chronic Kidney Disease

**Figure 1:**
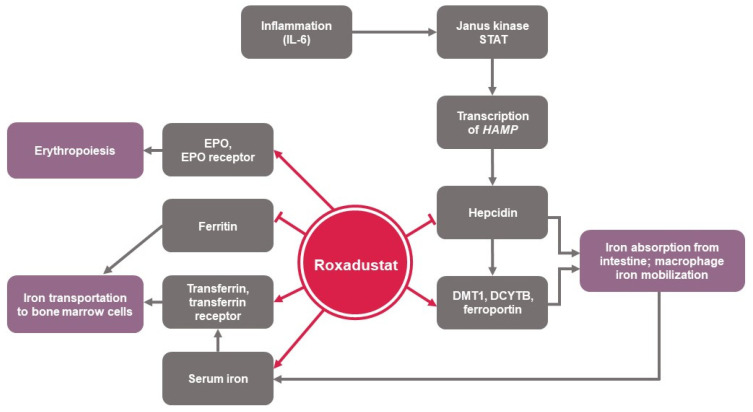
Effects of roxadustat on iron physiology and erythropoiesis [4]. DCYTB, duodenal cytochrome *b*; DMT1, divalent metal transporter 1; EPO, erythropoietin; IL-6, interleukin 6; STAT, signal transducer and activator of transcription.

Hypoxia-inducible factor (HIF) prolyl hydroxylase (PH) inhibitors represent a novel class of orally active small molecule drugs that transiently inhibit the HIF-PH enzymes, which causes the cellular accumulation of HIFs and induces activation of the genes involved in erythropoiesis, even in the presence of normal oxygen tension (i.e., transient “pseudohypoxic” state) [1]. HIF-PH inhibitors simulate hypoxia in erythropoietin-producing cells, stimulating endogenous erythropoietin synthesis and theoretically improving the supply of iron for erythropoiesis. Furthermore, HIF-PH inhibitors may reduce hepcidin levels, resulting in increased iron mobilization that may overcome functional iron deficiency associated with hyporesponsiveness to erythropoiesis-stimulating agents (ESAs) [1,2,3,4] (Figure 1).

Roxadustat has been evaluated for the treatment of anemia of chronic kidney disease (CKD) in sixteen phase 3 studies, including six studies in Japan [5,6,7,8,9], two studies in China [10,11], and eight studies that constitute the ALPINE Roxadustat Global Research Program [12,13,14,15,16,17,18,19] (Supplementary Table S1). In general, eligible patients were aged ≥18 years, were iron-replete with lower allowable values for ferritin and transferrin saturation (TSAT) in studies in patients with non-dialysis-dependent (NDD) CKD compared to patients with dialysis-dependent (DD) CKD, and had baseline hemoglobin levels between 9.0 and 12.0 g/dL and ≤10.0 g/dL for patients treated and untreated with an ESA, respectively [20,21]. In the NDD CKD trials [11,12,13,14], patients were randomized to receive either roxadustat or placebo thrice weekly (TIW), apart from one study in Japan (CL-0314) [7], which only randomized patients to different doses of roxadustat, and two studies primarily in Europe (DOLOMITES) [15] or in Japan (CL-0310) [6], which randomized patients to receive oral roxadustat or parenteral darbepoetin alfa (DA). In the DD CKD trials, patients received either oral roxadustat or a parenteral ESA (epoetin alfa [EA] or DA) [5,10,16,17,18,19], except in the three studies in Japan for which patients were randomized to different doses of roxadustat only [8,9]. Roxadustat starting doses were 50, 70, 100, 150, or 200 mg TIW, depending on the study, patient weight, and/or prior ESA exposure, and dosing was algorithmically adjusted throughout each study to achieve and/or maintain the target hemoglobin level [5,6,7,8,9,10,11,12,13,14,15,16,17,18,19].

Product labeling advises that patients should be iron-replete when roxadustat is initiated [22], a recommendation based on clinical experience and data. The percentage of patients who were iron-replete at baseline appeared to be similar between those randomized to roxadustat and those randomized to placebo or ESA [5,6,7,8,9,10,11,12,13,14,15,16,17,18,19] (Table 1).

Roxadustat is a novel agent with a unique mechanism of action compared to ESAs and a different combination of effects on iron parameters [22]. This narrative review describes the effects of treatment with roxadustat on iron parameters alongside iron supplementation and changes in hemoglobin in patients with anemia of NDD or DD CKD. Analyses that were published in manuscript or abstract form and were the primary, pooled, or post hoc analyses of phase 3 clinical studies evaluating roxadustat in patients with anemia of CKD were included.

### 1.2. Anemia of CKD and Iron Physiology

Anemia is a common complication of CKD, with a prevalence that increases with CKD severity and may be >50% in advanced stages of CKD [23,24]. Multiple mechanisms contribute to the development of anemia of CKD, including a relative and absolute erythropoietin deficiency, iron restriction secondary to inflammation and decreased hepcidin clearance, and absolute iron deficiency from gastrointestinal and iatrogenic blood loss during dialysis or blood draws, as well as shortened erythrocyte lifespan and inhibition of erythropoiesis by uremic toxins [24,25].

Iron is absorbed in the duodenum and bound to serum transferrin. Subsequently, iron is transported to the liver and spleen to be bound to ferritin for storage or to the bone marrow to be used in red blood cell production. Additionally, the iron regulatory hormone hepcidin regulates iron uptake from the gastrointestinal tract and release from macrophage and hepatocyte stores where iron is complexed with ferritin [24]. Ferroportin distributes iron between tissues, supporting iron absorption [26]. While erythropoiesis requires erythropoietin, iron also is required for hemoglobin synthesis and the differentiation of erythroblasts into reticulocytes [24]. Consequently, iron deficiency or iron restriction may cause resistance to erythropoietin [24]. Treatment of anemia of CKD with an ESA or another agent (e.g., a HIF-PH inhibitor) is individualized based on multiple factors, including hemoglobin concentration, anemia symptoms, prior response to iron therapy, stage of CKD, use of dialysis, and transfusion risk [27]. In general, various guidelines suggest treatment may be initiated in iron-replete patients with NDD CKD to reduce blood transfusions or when hemoglobin concentrations are <10.0 or <11.0 g/dL in symptomatic patients; treatment may be initiated when hemoglobin concentrations are 9.0–10.0 or <10.0 g/dL in patients with DD CKD, depending on the guideline. In both patient populations, it is recommended to avoid having hemoglobin concentrations fall below these values [27,28,29].

Erythropoietin production is stimulated by hypoxia through gene expression induced by HIFs [1,30]. At normal oxygen concentrations, a family of HIF-PHs hydroxylates the HIF-α subunit, resulting in its rapid proteasomal degradation [1]. As oxygen levels decrease, HIF-PH activity is lessened, and HIF-α accumulates, translocates into the nucleus, and dimerizes with HIF-β, activating transcriptional programs in response to hypoxia and enhancing erythropoiesis. Erythropoiesis results from increased production of erythropoietin and its receptor as well as from increases in various proteins needed for effective iron transport, absorption, and export from cells. HIFs function as master regulators of erythropoiesis by controlling not only the expression of the genes encoding erythropoietin and its receptor but also a battery of genes that regulate intestinal uptake, transport, release from internal stores, and bone marrow use of iron, which is required for hemoglobin synthesis [1].

Beginning in 1997, the Kidney Disease Outcomes Quality Initiative recommended that serum ferritin and TSAT be the primary tools for assessing iron management in patients with anemia of CKD [31]. A TSAT of ≤20% with an elevated serum ferritin level may serve as a harbinger of functional iron deficiency development from the supraphysiologic rate of red blood cell production when erythropoiesis is stimulated, which can result in a reduction in transferrin-bound circulating iron for incorporation into hemoglobin [31,32]. However, because TSAT is the ratio of serum iron to total iron-binding capacity (TIBC), the TSAT trend during treatment of anemia of CKD provides an incomplete understanding of the impact of treatment without taking into account changes in these component variables [24]. TIBC reflects serum transferrin level, because transferrin is the predominant carrier and binder of iron in blood plasma [24]. Additionally, other factors such as sampling time in relation to iron infusion time, type and dose of iron supplementation, inflammation, malnutrition, and chronic disease may affect TSAT, confounding its interpretation [24,31].

While the Kidney Disease: Improving Global Outcomes (KDIGO) guidelines and European Renal Best Practices (ERBP) statement for when a trial of intravenous (IV) iron may be suggested are similar (KDIGO: ferritin ≤ 500 µg/L, TSAT ≤ 30%; ERBP: NDD CKD ferritin < 200 µg/L, TSAT < 25% and DD CKD ferritin < 300 µg/L, TSAT < 25%), the decision in an individual patient may be affected by a variety of factors [27,28,29,33]. This likely is a result of considerable interpatient variability in ferritin levels, particularly because ferritin may be increased by inflammation and other non–iron-related factors, such as age, diabetes, nonalcoholic steatohepatitis, and obesity [21,33,34]. Following erythropoiesis stimulation in patients with CKD, serum hemoglobin increases, and serum ferritin sharply declines, indicating mobilization of iron stores for erythropoiesis [1,35]. The impact on serum iron can be variable and unpredictable at the individual patient level [33,34,35].

Hepcidin is the master regulator of systemic iron metabolism [36]. Hepcidin regulates dietary iron absorption and macrophage iron recycling from senescent red blood cells [25]. Iron overload and inflammation induce hepcidin synthesis [37], while iron deficiency, hypoxia, blood loss, and erythropoiesis stimulation inhibit hepcidin synthesis. Low plasma hepcidin concentration allows iron mobilization and iron use for erythropoiesis [36]. Elevated hepcidin levels can lead to iron-restricted erythropoiesis, due in part to reduced elemental iron absorption and reduced mobilization of iron from the reticuloendothelial system, and have been associated with increased mortality [25,38]. Because hepcidin can have a considerable effect on iron homeostasis and anemia of CKD, further elucidation of its trajectory over time in patients receiving roxadustat is necessary.

## 2. Results from Clinical Studies with Roxadustat in Patients with Anemia of CKD

Serum ferritin levels were evaluated in most of the clinical studies with roxadustat. Mean serum ferritin values decreased in patients with NDD and DD CKD randomized to receive roxadustat [5,6,7,8,9,10,11,12,13,14,15,16,17,18,19] (Table 1). Significantly greater reductions in serum ferritin occurred following roxadustat use in patients with NDD CKD compared to placebo use and in two of three studies in patients with DD CKD relative to ESA use [13,14,16,17,19]. In a pooled analysis of three studies in NDD CKD patients and three studies in DD CKD patients from the ALPINE program, in patients with NDD CKD, serum ferritin initially decreased through Week 8 following roxadustat initiation and gradually increased but remained below baseline values; there was no change in serum ferritin levels in the placebo group. In contrast, patients with DD CKD experienced an initial decrease in serum ferritin at Week 8 and subsequent gradual decreases in values through Week 52 that were similar to, though greater than, decreases seen with ESA [21]. In a post hoc analysis of patients in Japan with DD CKD, the mean doses of roxadustat per administration in the last 6 weeks of the study (Weeks 18–24) remained stable regardless of baseline markers of iron repletion (*p* = 0.208), whereas with DA, a significant increase in dosing was observed with decreasing ferritin (*p* < 0.001) [39]. In a similarly conducted post hoc analysis of patients in Japan with NDD CKD, patients with adequate iron repletion had the lowest doses for both roxadustat and DA [40].

Regional differences in baseline serum iron values were observed (e.g., in the European Union vs. in Japan) due to international differences in the standard of care [5,6,7,8,9,10,11,12,13,14,15,16,17,18,19] (Table 2). In the pooled analysis by Pergola et al., mean serum iron values initially decreased in patients with NDD CKD randomized to receive roxadustat before increasing above baseline by Week 20 and remaining relatively stable through Week 52, whereas patients randomized to placebo had minimal or no change in serum iron values. However, oral iron administration was not restricted in the NDD CKD population, and information about oral iron use and dose was incompletely collected [21]. In patients with DD CKD randomized to roxadustat, mean serum iron values increased slightly from baseline and remained relatively stable through Week 52; however, patients randomized to ESA experienced a slight decrease in serum iron values that was significant compared to values in patients treated with roxadustat [16,17,19] (Table 2). This decrease in mean serum iron occurred by Week 8, after which values remained relatively stable [21].

In patients treated with roxadustat, TIBC increased absolutely and relative to placebo and ESA comparators in the NDD and DD CKD populations [5,6,7,8,9,10,11,12,13,14,15,16,17,18,19] (Supplementary Table S2). Results from the pooled analyses described by Pergola et al. suggest the increase in TIBC occurred by Week 8 in patients with NDD or DD CKD and remained largely unchanged through Week 52 [21]. Additionally, most of the studies that evaluated TSAT found a slight decrease in patients randomized to receive roxadustat, although this decrease was not statistically significant compared to placebo or ESA [5,6,7,8,9,10,11,12,13,14,15,16,17,18,19] (Supplementary Table S3). When evaluating patients with NDD CKD [21], patients receiving roxadustat had a decrease in TSAT at Week 8 that was no longer observed by Week 52, whereas patients who received placebo showed no change in TSAT throughout treatment. However, in patients with DD CKD, an average decrease in TSAT of approximately 10% was observed, and the reduced TSAT was maintained through Week 52 in patients randomized to roxadustat or ESA [21].

In the pooled analysis performed by Pergola et al., the adjusted least squares mean (LSM) change in hemoglobin levels from baseline averaged over Weeks 28–52 was greater with roxadustat versus placebo in the NDD CKD population (LSM difference: 1.7 [95% CI: 1.7, 1.8]), with a less-pronounced difference in the DD CKD population when compared to ESA (LSM difference: 0.3 [95% CI: 0.2, 0.3]) [21]. This finding was also observed in the NDD/incident-to-dialysis (ID)-DD population when roxadustat was compared to ESA in a pooled analysis of four ALPINE studies (LSM difference: 0.20 [95% CI: 0.038, 0.362]) [15,16,17,19,41]. In a pooled analysis of three ALPINE studies in NDD CKD, the improvement in hemoglobin from baseline was 1.94 g/dL in both the iron-replete and iron-depleted groups [12,13,14,42]. Finally, in a pooled analysis of the four ALPINE studies in DD CKD, hemoglobin change from baseline to Weeks 28–36 achieved noninferiority for roxadustat versus ESA in each study and in the ID-DD (LSM difference: 0.28 [95% CI: 0.11, 0.45]) and stable dialysis (LSM difference: 0.30 [95% CI: 0.23, 0.37]) subgroups [20].

Additionally, the correction and maintenance of serum hemoglobin is affected by iron supplementation. To increase hemoglobin 1 g/dL, approximately 170 mg of iron is required based on a total blood volume of 4.9 L in a 70 kg man [43]. Across the NDD, ID-DD, and DD CKD subpopulations, the proportion of patients who received IV iron supplementation at Week 52 was lower for those randomized to roxadustat compared to those randomized to ESA or placebo (NDD/ID-DD 36.5% vs. 49.1%) [21,41]. In the subpopulation of patients with DD CKD, the mean amount of IV iron provided was numerically lower with roxadustat versus ESA in both ID-DD (80.2% vs. 81.8%) and stable DD patients (89.2% vs. 93.5%) [20,41].

In general, hepcidin levels decreased in patients who received roxadustat compared to baseline values in all CKD populations [5,6,7,8,9,10,11,12,13,14,15,16,17,18,19] (Table 3). These decreases appear to be more robust with roxadustat than with ESA or placebo. The baseline hepcidin values in quartiles were evaluated by Pergola et al. to determine the change from baseline in patients randomized to receive roxadustat or placebo (in NDD CKD patients) or ESA (in DD CKD patients) [21]. As the baseline hepcidin value increased, there was more suppression of hepcidin from baseline for patients with NDD or DD CKD who were randomized to receive roxadustat. Patients with NDD CKD randomized to receive placebo had an increase in mean hepcidin values in all but the highest quartile. Patients with DD CKD randomized to receive ESA had a less substantial decrease in mean hepcidin values compared to roxadustat recipients in each quartile. Similar findings have been observed by Lei and colleagues [44].

## 3. Interpretation of Changes in Iron Parameters

The rate of decline in ferritin levels has previously been shown to correspond to the extent of erythropoiesis stimulation, reflecting the shift of iron from stores into erythrocyte hemoglobin [45] (Figure 1). Roxadustat was associated with a more pronounced reduction in serum ferritin and an increase in serum iron compared to a decrease in both parameters with ESA. This suggests that roxadustat promoted the release of sequestered iron from intracellular stores for incorporation into erythrocyte progenitors. Notably, potential differences between patients with NDD CKD versus DD CKD in changes in serum ferritin could have been affected by differences in the use of oral and IV iron supplementation, as well as underlying pathophysiological differences between NDD and DD CKD. Potential sequelae from high body iron stores (i.e., toward the upper limit of normal values) require further investigation [46]. While roxadustat likely increases iron absorption and mobilization, current product labeling and expert opinion advise that patients should be iron-replete when roxadustat is initiated [22].

While the decreases in TSAT in patients treated with roxadustat were relatively small and, in the case of patients with NDD CKD, not observed by Week 52, these changes reflect the concomitant increases in both serum iron and TIBC. In particular, TIBC rose more quickly than serum iron, which remained generally stable through Week 8 in patients with NDD CKD, suggesting that a relative iron deficiency does not commonly develop in patients receiving roxadustat because of iron being mobilized and utilized following roxadustat initiation [1]. Observed differences in TIBC and TSAT were predominately seen in the initiation stage of treatment; however, because iron supplementation was allowed, the effect on the total iron supply is uncertain. After an initial decrease in serum ferritin that stabilized, iron mobilization may reach an equilibrium and increases in serum iron thereafter may be more impacted by iron absorption and iron supplementation. The stimulatory effect of roxadustat on TIBC and transferrin may result from the presence of a hypoxia regulatory element in the transferrin gene [47]. Notably, these data for TSAT, TIBC, and serum iron levels suggest that the mechanisms behind the similar changes in TSAT over time in patients randomized to roxadustat or an ESA appear to be different. While this is a distinguishing factor for roxadustat compared to ESAs, the clinical impact of this difference must still be evaluated.

Increased erythropoiesis exerts negative feedback on hepcidin synthesis, but hepcidin is not a direct target of HIF stabilization. Nevertheless, roxadustat does appear to have some additional direct and/or indirect effects on hepcidin beyond those seen with ESAs [36]. A potential mechanism for this additional decrease in hepcidin in patients treated with roxadustat includes the effect of stimulation of transferrin synthesis. Of the four major forms of transferrin, diferric, N-lobe monoferric, C-lobe monoferric, and apotransferrin, diferric transferrin is believed to be the most potent stimulus for hepcidin production. Higher transferrin concentrations diminish the concentration of diferric transferrin as iron redistributes to monoferric forms, which are less effective at stimulating hepcidin [48]. Additionally, the extent to which iron supplementation, both oral and IV, affects hepcidin values requires further elucidation. As an increased risk of venous thromboembolism has been associated with higher plasma hepcidin levels, further investigation into the potential sequelae and clinical implications from effects of roxadustat on hepcidin is needed [46].

The effects from oral and IV iron supplementation and blood transfusions throughout the studies must be considered. While these findings may suggest improved iron availability and increased erythropoiesis while requiring less IV iron use with roxadustat versus comparators and are consistent with the mechanism of action for roxadustat, they should be interpreted cautiously. Most notably, in the ROCKIES study, IV iron was restricted to rescue therapy in the roxadustat group, whereas IV iron was administered per local guidelines in the ESA arm [17]. However, in the other ALPINE studies in DD CKD, which comprise most patients, IV iron could be part of the supplementation regimen, al-though criteria for use may have been more stringent in the roxadustat group (e.g., hemoglobin level had not responded adequately to roxadustat following two consecutive dose increases or in patients who had reached the maximum dose limit, and patients’ ferritin level was <100 µg/L, or TSAT was <20%, or patients were intolerant of oral iron therapy) compared to ESA (given according to standard of care) [21]. In ALPINE studies in NDD CKD, oral iron was administered without restriction in the roxadustat and placebo arms, although information on use and dose was incompletely collected. Though there were regional differences in iron supplementation use due to differences in the standard of care (e.g., in the European Union vs. Japan vs. the United States), in general, roxadustat appears to promote iron availability and utilization while not precipitating an iron-deficient state in the average patient.

## 4. Clinical Considerations

Roxadustat’s mechanism of action on erythropoiesis differs from that of ESAs, resulting in unique and potentially beneficial effects on iron stores and availability. These differences should be considered holistically while treating patients with anemia of NDD and DD CKD. The use of supplemental IV iron is generally decreased in patients receiving roxadustat, likely due to an increased promotion of iron availability and utilization. This finding should not preclude clinicians from following best practices for monitoring of iron parameters or providing iron supplementation. However, this reduced use of IV iron, particularly in the NDD CKD population, may spare veins that could be viable vascular access sites in the future if the patient progresses to requiring dialysis. The outcomes in the average iron-deplete patients receiving roxadustat are encouraging; however, current product labeling and expert opinion advise that patients should be iron-replete when roxadustat is initiated and continued [22]. Additionally, the beneficial effects of roxadustat on iron parameters should be considered alongside efficacy and safety outcomes. Pooled analyses in patients with DD CKD receiving roxadustat have established a safety profile comparable to the safety profile of ESAs and noninferior risk of major adverse cardiovascular events in patients with NDD CKD compared to placebo.

## 5. Conclusions

Less IV iron supplementation is generally used in patients with NDD CKD treated with roxadustat, an oral medication, than in patients who received placebo, and in patients with DD CKD treated with roxadustat than in patients treated with an ESA, while achieving noninferior (vs. ESA) and superior (vs. placebo) effects on hemoglobin change from baseline. The distinctive effects of roxadustat on iron physiology were generally similar in direction though not necessarily in the extent of change in patients with NDD and DD CKD (decreased serum ferritin, TSAT, and hepcidin and increased serum iron and TIBC) and when compared to ESAs may decrease the use of iron to support erythropoiesis. The extent to which factors such as iron supplementation and study drug dose affected iron parameters requires further investigation.

## Figures and Tables

**Table 1:** Serum ferritin levels in patients with chronic kidney disease randomized to receive roxadustat, erythropoiesis-stimulating agent (ESA), or placebo (µg/L).

Study	Roxadustat			ESA			Placebo			LSMD (95% CI)	*p* Value
	*n*	Mean Baseline	LSM CFB (95% CI)	*n*	Mean Baseline	LSM CFB (95% CI)	*n*	Mean Baseline	LSM CFB (95% CI)		
Chen [11]	85	191.4	−93.3 (146.3) ^a^	-	-	-	43	271.1	−21.9 (115.5) ^a^	−102.2 (−142.6, −61.7)	-
Chen [10]	BL: 204 Wk 27: 160	498.5	−99	BL: 100 Wk 27: 94	420.1	−133	-	-	-	35 (−12, 82)	-
CL-0314 [7]	99	113.8	−27.4 (46.4) ^a^	-	-	-	-	-	-	-	-
CL-0310 [6]	201	142.6	-	131	150.4	-	-	-	-	-	-
CL-0307 [5]	150	102.3	−4.0 (78.4) ^a^	151	96.3	−18.8 (64.6) ^a^	-	-	-	-	-
CL-0308 [9]	74	127.1	−74.3 (68.3) ^a^	-	-	-	-	-	-	-	-
CL-0312 [9]	163	108.3	−23.99 (76.3) ^a^	-	-	-	-	-	-	-	-
CL-0302 [8]	ESA-N: 13 ESA-C: 43	ESA-N: 269.6 ESA-C: 145.4	ESA-N: −110.5 (80.9) ^a^ ESA-C: −37.5 (83.1) ^a^	-	-	-	-	-	-	-	-
ALPS ^b^	BL: 389 Wk 52: 276	241.2	−73.0 (251.2) ^a^	-	-	-	BL: 203 Wk 52: 118	264.7	41.5 (285.2) ^a^	-	-
OLYMPUS [13]	BL: 1384 Wk 24: 1200	248.3	−37.1 (−51.1, −23.1)	-	-	-	BL: 1377 Wk 24: 1050	241.4	17.5 (2.9, 32.1)	−54.5 (−71.7, −37.4)	<0.001
ANDES [14]	BL: 607 Wk 44: 478	306.9	−44.6 (−68.9, −20.3)	-	-	-	BL: 305 Wk 44: 187	308.4	12.9 (−20.4, 46.3)	−57.5 (−92.8, −22.3)	0.0014
DOLOMITES [15]	323	233.8	−41.4 (232.0) ^a^	293	225.0	−32.2 (204.4) ^a^	-	-	-	-	-
HIMALAYAS [16]	BL: 522 Wk 44: 362	441.4	−191.3 (−234.4, −148.2)	BL: 513 Wk 44: 381	436.7	−130.0 (−172.9, −87.2)	-	-	-	−61.3 (−117.0, −5.6)	0.031
ROCKIES [17]	BL: 1051 Wk 24: 875	543.0	−104.5 (−126.2, −82.8)	BL: 1055 Wk24: 946	555.8	−41.2 (−62.1, −20.3)	-	-	-	−63.3 (−87.4, −39.2)	<0.001
PYRENEES ^b^	BL: 413 Wk 52: 318	636.3	−273.8 (301.7) ^a^	BL: 420 Wk 52: 365	733.8	−154.7 (471.2) ^a^	-	-	-	-	-
SIERRAS [19]	BL: 369 Wk 52: 238	445.9	−198.0 (−224.4, −180.5)	BL: 370 Wk 52: 273	426.9	−183.9 (−205.2, −192.6)	-	-	-	−18.6 (−43.0, 5.8)	0.14

Note: n numbers are the same for baseline and CFB unless otherwise specified. CFB, change from baseline; ESA, erythropoiesis-stimulating agent; ESA-N, ESA-naive; ESA-C, ESA-converted; LSM, least squares mean; LSMD, least squares mean difference. ^a^ Change from baseline (standard deviation) rather than least squares mean change from baseline. ^b^ Astellas data on file.

**Table 2:** Serum iron levels in patients with chronic kidney disease randomized to receive roxadustat, erythropoiesis-stimulating agent (ESA), or placebo (µmol/L).

Study	Roxadustat			ESA			Placebo			LSMD (95% CI)	*p* Value
	*n*	Mean Baseline	LSM CFB (95% CI)	*n*	Mean Baseline	LSM CFB (95% CI)	*n*	Mean Baseline	LSM CFB (95% CI)		
Chen [11]	85	10.7	−0.2 (6.3) ^a^	-	-	-	43	11.4	−0.6 (4.4) ^a^	0.2 (−1.7, 2.2)	-
Chen [10]	BL: 204 Wk 27: 160	15.1	0.6	BL: 100 Wk 27: 94	14.3	−3.9	-	-	-	4.4 (3.0, 5.9)	-
CL-0314 [7]	99	13.3	2.0 (5.5) ^a^	-	-	-	-	-	-	-	-
CL-0310 [6]	201	16.0	-	131	15.0	-	-	-	-	-	-
CL-0307 [5]	150	12.1	1.2 (6.4) ^a^	151	12.6	−0.9 (5.5) ^a^	-	-	-	-	-
CL-0308 [9]	74	11.9	0.4 (6.6) ^a^	-	-	-	-	-	-	-	-
CL-0312 [9]	163	12.7	0.4 (7.4) ^a^	-	-	-	-	-	-	-	-
CL-0302 [8]	ESA-N: 13 ESA-C: 43	ESA-N: 18.4 ESA-C: 17.7	ESA-N: −3.1 (6.4) ^a^ ESA-C: −2.0 (7.2) ^a^	-	-	-	-	-	-	-	-
ALPS [12] ^b^	-	-	-	-	-	-	-	-	-	-	-
OLYMPUS [13]	BL: 1384 Wk 24: 1201	12.1	1.2 (0.9, 1.5)	-	-	-	BL: 1377 Wk 24: 1050	12.0	−0.2 (−0.5, 0.1)	1.4 (1.0, 1.7)	<0.001
ANDES [14]	BL: 608 Wk 44: 480	11.7	1.9 (1.2, 2.5)	-	-	-	BL: 305 Wk 44: 188	11.9	0.4 (−0.6, 1.3)	1.5 (0.5, 2.4)	0.0026
DOLOMITES [15]	BL: 322 Wk 52: 253	11.3	1.1 (5.9) ^a^	BL: 292 Wk 52: 243	10.7	2.2 (6.2) ^a^	-	-	-	-	-
HIMALAYAS [16]	BL: 522 Wk 44: 364	11.5	0.4 (−0.2, 1.0)	BL: 513 Wk 44: 384	11.0	−0.8 (−1.4, −0.3)	-	-	-	1.2 (0.4, 2.0)	0.0024
ROCKIES [17]	BL: 1051 Wk 24: 877	13.4	1.2 (0.8, 1.6)	BL: 1055 Wk 24: 946	13.2	−1.0 (−1.4, −0.6)	-	-	-	2.2 (1.8, 2.6)	<0.001
PYRENEES ^c^	BL: 414 Wk 52: 316	12.6	−0.3 (7.4) ^a^	BL: 257 Wk 52: 221	12.4	−1.3 (6.3) ^a^	-	-	-	-	-
SIERRAS [19]	BL: 369 Wk 52: 238	12.0	0.2 (−0.5, 1.0)	BL: 370 Wk 52: 273	12.1	−2.2 (−2.9, −1.6)	-	-	-	2.4 (1.6, 3.3)	<0.0001

Note: n numbers are the same for baseline and CFB unless otherwise specified. CFB, change from baseline; ESA, erythropoiesis-stimulating agent; ESA-N, ESA-naive; ESA-C, ESA-converted; LSM, least squares mean; LSMD, least squares mean difference. ^a^ Change from baseline (standard deviation) rather than least squares mean change from baseline. ^b^ Source provides no values for this parameter. ^c^ Astellas data on file.

**Table 3:** Hepcidin levels in patients with chronic kidney disease randomized to receive roxadustat, erythropoiesis-stimulating agent (ESA), or placebo (ng/mL) ^a^.

Study	Roxadustat			ESA			Placebo			LSMD (95% CI)	*p* Value
	*n*	Mean Baseline	LSM CFB (95% CI)	*n*	Mean Baseline	LSM CFB (95% CI)	*n*	Mean Baseline	LSM CFB (95% CI)		
Chen [11]	86	95.9	−56.1 (63.4) ^b^	-	-	-	44	114.7	−15.1 (48.1) ^b^	−49.8 (−66.8, −32.8)	-
Chen [10]	BL: 204 Wk 27: 155	180.7	−30.2 (−64.8, −13.6)	BL: 100 Wk 27: 90	148.3	−2.3 (−51.6, 6.2)	-	-	-	-	-
CL-0314 [7]	99	24.0	−5.7 (18.4) ^b^	-	-	-	-	-	-	-	-
CL-0310 [6]	201	39.3	-	131	39.7	-	-	-	-	-	-
CL-0307 ^c^	150	26.4	2.3 (27.3) ^b^	151	24.4	−0.6 (27.1) ^b^	-	-	-	-	-
CL-0308 [9]	74	39.8	−23.2 (22.8) ^b^	-	-	-	-	-	-	-	-
CL-0312 [9]	163	28.8	−6.2 (29.0) ^b^	-	-	-	-	-	-	-	-
CL-0302 [8]	ESA-N: 13 ESA-C: 43	ESA-N: 63.6 ESA-C: 45.9	ESA-N: −31.8 (39.4) ^b^ ESA-C: −19.0 (21.6) ^b^	-	-	-	-	-	-	-	-
ALPS [12]	BL: 382 Wk 52: 268	37.9	−12.3 (37.4) ^b^	-	-	-	BL: 195 Wk 52: 114	41.2	−2.0 (40.7) ^b^	-	-
OLYMPUS [13]	BL: 1384 Wk 24: 658	163.2	−35.9 (−44.9, −27.0)	-	-	-	BL: 1377 Wk 24: 604	155.5	9.4 (0.2, 18.7)	−45.4 (−56.2, −34.5)	<0.001
ANDES [14]	BL: 608 Wk 44: 396	110.7	−18.8 (−27.4, −10.3)	-	-	-	BL: 305 Wk 44: 152	106.1	6.9 (−5.3, 19.1)	−25.7 (−38.5, −12.9)	<0.0001
DOLOMITES [15] ^d^	-	-	-	-	-	-	-	-	-	-	-
HIMALAYAS [16]	BL: 522 Wk 44: 356	173.2	−64.8 (−74.3, −55.3)	BL: 513 Wk 44: 372	169.9	−54.1 (−63.4, −44.7)	-	-	-	−10.7 (−23.2, 1.8)	0.093
ROCKIES [17]	BL: 1051 Wk 24: 608	275.6	−45.0 (−57.5, −32.5)	BL: 1055 Wk 24: 625	269.6	−16.8 (−29.2, −4.4)	-	-	-	−28.2 (−42.0, −14.5)	<0.001
PYRENEES ^c^	404	73.4	−32.7 (42.3) ^b^	413	80.8	−17.5 (47.3) ^b^	-	-	-	-	-
SIERRAS [19]	BL: 369 Wk 52: 230	272.9	−99.9 (−118.2, −81.6)	BL: 370 Wk 52: 265	270.7	−80.8 (−98.3, −63.3)	-	-	-	−19.1 (−39.5, 1.3)	0.066

Note: n numbers are the same for baseline and CFB unless otherwise specified. CFB, change from baseline; ESA, erythropoiesis-stimulating agent; ESA-N, ESA-naive; ESA-C, ESA-converted; LSM, least squares mean; LSMD, least squares mean difference. ^a^ To convert to SI units (nmol/L), multiply numbers by 0.3584. ^b^ Change from baseline (standard deviation) rather than least squares mean change from baseline. ^c^ Astellas data on file. ^d^ Source provides no values for this parameter.

## Data Availability

Researchers may request access to anonymized participant level data, trial level data, and protocols from Astellas sponsored clinical trials at www.clinicalstudydatarequest.com. For the Astellas criteria on data sharing see: https://clinicalstudydatarequest.com/Study-Sponsors/Study-Sponsors-Astellas.aspx.

## Supplementary Materials

The following information can be downloaded at: https://www.mdpi.com/article/10.3390/jcm12134217/s1, Table S1: Study Characteristics of Phase 3 Trials in Patients With Non–Dialysis-Dependent (NDD) and Dialysis-Dependent (DD) Chronic Kidney Disease Randomized to Receive Roxadustat; Table S2: Total Iron-Binding Capacity in Patients With Chronic Kidney Disease Randomized to Receive Roxadustat, Erythropoiesis-Stimulating Agent (ESA), or Placebo (µg/dL); Table S3: Transferrin Saturation in Patients With Chronic Kidney Disease Randomized to Receive Roxadustat, Erythropoiesis-Stimulating Agent (ESA), or Placebo (%).
