# Supplementary material for: Iron Parameters in Patients Treated with Roxadustat for Anemia of Chronic Kidney Disease
Source: J Clin Med. 2023 Jun 22;12(13):4217. doi: 10.3390/jcm12134217 (PMC10342436; doi:10.3390/jcm12134217)
Supplement: Supplementary file 1 [file jcm-12-04217-s001.zip › jcm-2311282-supplementary.pdf]

Iron Parameters in Patients Treated with Roxadustat for Anemia of Chronic Kidney Disease
Tomas Ganz, Francesco Locatelli, Mustafa Arici, Tadao Akizawa, and Michael Reusch

Supplementary Material

Table S1. Study Characteristics of Phase 3 Trials in Patients With Non–Dialysis-Dependent (NDD) and Dialysis-Dependent (DD) Chronic Kidney Disease Randomized to Receive Roxadustat

Table S2. Total Iron-Binding Capacity in Patients With Chronic Kidney Disease Randomized to Receive Roxadustat, Erythropoiesis-Stimulating Agent (ESA), or Placebo (µg/dL)[a]

Table S3. Transferrin Saturation in Patients With Chronic Kidney Disease Randomized to Receive Roxadustat, Erythropoiesis-Stimulating Agent (ESA), or Placebo (%)

Table S1. Study Characteristics of Phase 3 Trials in Patients With Non–Dialysis-Dependent (NDD) and Dialysis-Dependent (DD) Chronic Kidney Disease Randomized to Receive Roxadustat

	**Chen[11]**	**Chen[10]**	**CL-0314[7]**	**CL-0310**[6]	**CL-0307[5]**	**CL-0308[9]**	**CL-0312[9]**	**CL-0302[8]**	**ALPS**[12]	**OLYMPUS[13]**	**ANDES**[14]	**DOLOMITES[15]**	**HIMALAYAS**[16]	**ROCKIES[17, 20]**	**PYRENEES**[18]	**SIERRAS[19]**
Region	China	China	Japan	Japan	Japan	Japan	Japan	Japan	Global	Global	Global	Europe	Global	Global	Europe	United States
Comparator	PBO	EA	–	DA	DA	–	–	–	PBO	PBO	PBO	DA	EA	EA	DA or EA	EA
Randomized, *n*	154	305	100	334	303	75	164[a]	ESA-N: 13<br>ESA-C: 43	597	2781	922	616	1043	2106	838	741
NDD, *n*	154	–	100	334	–	–	–	–	597	2781	922	616	NA	NA	NA	NA
ID-DD, *n*	–	–	–	–	–	75		56	–	–	–	NA	1039	283	–	71
Stable DD, *n*	–	305	–	–	303		164		–	–	–	NA		1823	838	
Baseline Hb, g/dL[b,c]	7.0-10.0	9.0-12.0	<10.5	10.0-12.0	10.0-12.0	≤10.0	10.0-12.0	ESA-N: <10.5<br>ESA-C: 10.0-12.0	≤10.0	<10.0	≤10.0	≤10.5	≤10.0	<10.0 or <12.0	9.5-12.0	9.0-12.0
Hb target, g/dL[c]	100-120	100-120	100-120	100-120	100-120	100-120	100-120	100-120	100-120	≥110	≥110	100-120	100-120[d]	100-120[d]	100-120	100-120[d]
Roxadustat starting dose, mg, TIW	70 (pts 40-60 kg)<br>100 (pts ≥60 kg)	100 (pts 45-60 kg)<br>120 (pts ≥60 kg)	50 or 70	70 or 100	70 or 100	50 or 70	70 or 100	ESA-N: 50 or 70<br>ESA-C: 70 or 100	70 (pts 45-70 kg)<br>100 (pts 70-160 kg)	70	70 (pts 45-70 kg)<br>100 (pts ≥70 kg)	70 or 100	70 (pts ≤70 kg)<br>100 (pts 70-160 kg)	70 (pts 45-70 kg)<br>100 (pts 70-160 kg)	100, 150, or 200[e]	70, 100, 150, or 200[e]
Baseline characteristics																
Iron-replete, *n* (%)	–	–	43 (43.4)	106 (52.7)	44 (29.1)	–	–	ESA-N: 11 (84.6)<br>ESA-C: 19 (44.2)	204 (52.2)	809 (58.5)	373 (60.6)	182 (56.3)	406 (77.8)	–	355 (86.0)	360 (97.3)
Age, y, mean (SD)	54.7 (13.3)	47.6 (11.7)	68.8 (11.3)	69.3 (11.2)	64.6 (11.7)	66.2 (12.1)	62.8 (11.8)	ESA-N: 66.1 (10.9)<br>ESA-C: 63.7 (10.1)	62.0[f] (20-89)	60.9 (14.7)	64.9 (12.6)	66.8 (13.6)	53.8 (14.7)	53.5 (15.3)	61.0 (13.8)	57.6 (13.6)
eGFR, mL/min, mean (SD)	16.5 (8.0)	–	–	17.7 (7.9)	–	–	–	–	16.5 (10.2)	19.7 (11.7)	21.9 (11.5)	20.3 (11.5)	–	–	–	–
Dialysis vintage (y), mean (SD)	–	4.5 (3.5)	–	–	7.7 (7.5)	0.053 (0.051)	7.5 (6.6)	ESA-N: 3.0 (3.2)<br>ESA-C: 3.2 (3.2)	–	–	–	–	0.2 (0.07)	–	4.4 (4.2)	4.0 (3.5)

DA, darbepoetin alfa; EA, epoetin alfa; eGFR, estimated glomerular filtration rate; ESA, erythropoiesis-stimulating agent; ESA-N, ESA-naive; ESA-C, ESA-converted; Hb, hemoglobin; ID-DD, incident-to-dialysis dialysis-dependent; NA, not applicable or not available; PBO, placebo; pts, patients; SD, standard deviation; TIW, 3 times per week.
[a]Patients were registered to 1 or 2 roxadustat groups, not randomized.
[b]Baseline hemoglobin varied by ESA use at time of randomization.
[c]To convert to SI units (g/L), multiply numbers by 10.
[d]Hemoglobin maintenance target in ESA-treated patients followed local guidelines and labeling.
[e]Dependent on initial ESA dose 4 weeks prior to randomization.
[f]Median (range).

Table S2. Total Iron-Binding Capacity in Patients With Chronic Kidney Disease Randomized to Receive Roxadustat, Erythropoiesis-Stimulating Agent (ESA), or Placebo (µg/dL)[a]

	Roxadustat			ESA			Placebo				
**Study**	***n***	**Mean Baseline**	**LSM CFB (95% CI)**	***n***	**Mean Baseline**	**LSM CFB (95% CI)**	***n***	**Mean Baseline**	**LSM CFB (95% CI)**	**LSMD (95% CI)**	***P* Value**
Chen[11]	85	305.25	101.68 (66.82)[b]	–	–	–	43	290.95	-1.84 (54.30)[b]	105.53 (82.63, 128.38)	–
Chen[10]	BL: 204 Wk 24: 160	264.80	53.07	BL: 100 Wk 24: 94	269.83	-6.70	–	–	–	59.78 (45.25, 74.30)	–
CL-0314[7]	99	268.72	32.96 (40.22)[b]	–	–	–	–	–	–	–	–
CL-0310[6]	201	265.92	–	131	259.78	–	–	–	–	–	–
CL-0307**[5]**	150	242.46	43.58 (45.25)[b]	151	243.02	8.94 (31.84)[b]	–	–	–	–	–
CL-0308[9]	74	236.3	75.4 (60.9)[b]	–	–	–	–	–	–	–	–
CL-0312[9]	163	248.6	55.9 (53.6)[b]	–	–	–	–	–	–	–	–
CL-0302[8]	–	–	–	–	–	–	–	–	–	–	–
ALPS[12][c]	–	–	–	–	–	–	–	–	–	–	–
OLYMPUS**[13]**	BL: 1384 Wk 24: 1200	230.44	30.79 (28.08, 33.50)	–	–	–	BL: 1377 Wk 24: 1050	232.46	-3.82 (-6.65, -0.99)	34.61 (31.29, 37.93)	<0.001
ANDES[14]	BL: 608 Wk 44: 476	257.18	35.07 (30.13, 40.01)	–	–	–	BL: 305 Wk 44: 185	262.30	-3.58 (-10.33, 3.17)	38.65 (31.86, 45.45)	<0.0001
DOLOMITES[15][c]	–	–	–	–	–	–	–	–	–	–	–
HIMALAYAS[16]	BL: 522 Wk 44: 364	241.04	37.70 (33.28, 42.12)	BL: 513 Wk 44: 383	238.06	1.65 (-2.73, 6.02)	–	–	–	36.05 (30.23, 41.87)	<0.0001
ROCKIES**[17]**	BL: 1051 Wk 24: 874	208.16	34.98 (31.77, 38.18)	BL: 1055 Wk 24: 946	208.64	-2.41 (-5.50, 0.68)	–	–	–	37.38 (33.82, 40.95)	<0.001
PYRENEES[18]	–	–	55.75 (49.38)[b]	–	–	15.31 (35.70)[b]	–	–	–	–	–
SIERRAS**[19]**	BL: 369 Wk 52: 235	201.88	47.21 (43.06, 51.37)	BL: 370 Wk 52: 272	202.89	10.94 (6.95, 14.92)	–	–	–	36.28 (31.73, 40.82)	<0.0001

Note: n numbers are the same for baseline and CFB unless otherwise specified.
CFB, change from baseline; LSM, least squares mean; LSMD, least squares mean difference.
[a]To convert to SI unit (µmol/L), multiply numbers by 0.179.
[b]Change from baseline rather than least squares mean change from baseline.
[c]Source provides no values for this parameter.

Table S3. Transferrin Saturation in Patients With Chronic Kidney Disease Randomized to Receive Roxadustat, Erythropoiesis-Stimulating Agent (ESA), or Placebo (%)

	**Roxadustat**			**ESA**			**Placebo**				
**Study**	***n***	**Mean Baseline**	**LSM CFB (95% CI)**	***n***	**Mean Baseline**	**LSM CFB (95% CI)**	***n***	**Mean Baseline**	**LSM CFB (95% CI)**	**LSMD (95% CI)**	***P* Value**
Chen[11]	85	20.6	-5.2 (10.4)[a]	–	–	–	43	23.2	-1.7 (9.2)[a]	-4.3<br>(-7.4, -1.1)	–
Chen[10]	BL: 204<br>Wk 27: 160	33.8	-4.5	BL: 100<br>Wk 27: 94	30.0	-8.7	–	–	–	4.2<br>(1.5, 6.9)	–
CL-0314[7]	99	27.9	1.0 (10.5)[a]	–	–	–	–	–	–	–	–
CL-0310[6]	201	34.1	–	131	32.7	–	–	–	–	–	–
CL-0307**[5]**	150	28.3	-1.1 (13.8)[a]	151	29.0	-2.4 (13.8)[a]	–	–	–	–	–
CL-0308[9]	74	28.5	-5.0 (14.8)[a]	–	–	–	–	–	–	–	–
CL-0312[9]	163	29.1	-3.9 (15.4)[a]	–	–	–	–	–	–	–	–
CL-0302[8]	ESA-N: 13<br>ESA-C: 43	ESA-N: 45.8<br>ESA-C: 35.9	ESA-N: -14.6 (15.8)[a]<br>ESA-C: -8.1 (14.1)[a]	–	–	–	–	–	–	–	–
ALPS[12],[b]	–	–	–	–	–	–	–	–	–	–	–
OLYMPUS**[13]**	BL: 1384<br>Wk 224: 1199	29.9	-0.8<br>(-1.4, -0.2)	–	–	–	BL: 1377<br>Wk 24: 1047	29.5	-0.3<br>(-0.9, 0.4)	-0.6<br>(-1.3, 0.2)	0.13
ANDES[14]	BL: 606<br>Wk 44: 476	26.3	0.4<br>(-0.8, 1.7)	–	–	–	BL: 304<br>Wk 44: 185	26.2	0.5<br>(-1.2, 2.3)	-0.1<br>(-2.0, 1.7)	0.90
DOLOMITES[c]	323	24.0	1.3 (11.8)[a]	293	23.3	5.2 (13.2)[a]	–	–	–	–	–
HIMALAYAS[16]	BL: 522<br>Wk 44: 364	27.0	-2.7<br>(-3.9, -1.5)	BL: 513<br>Wk 44: 383	27.6	-2.2<br>(-3.4, -1.1)	–	–	–	-0.5<br>(-2.0, 1.1)	0.54
ROCKIES**[17]**	BL: 1051<br>Wk 24: 866	36.0	-1.9<br>(-2.8, -1.1)	BL: 1055<br>Wk 24: 939	35.0	-2.4<br>(-3.3, -1.6)	–	–	–	0.5<br>(-0.4, 1.5)	0.29
PYRENEES[c]	413	33.4	-7.3 (17.2)[a]	257	33.2	-5.7 (15.0)[a]	–	–	–	–	–
SIERRAS**[19]**	BL: 369<br>Wk 52: 236	33.6	-6.6<br>(-8.4, -4.8)	BL: 370<br>Wk 52: 272	33.7	-8.8<br>(-10.5, -7.0)	–	–	–	2.2<br>(0.2, 4.2)	0.034

Note: n numbers are the same for baseline and CFB unless otherwise specified.

CFB, change from baseline; ESA, erythropoiesis-stimulating agent; ESA-N, ESA-naive; ESA-C, ESA-converted; LSM, least squares mean; LSMD, least squares mean difference.

[a]Change from baseline (standard deviation) rather than least squares mean change from baseline.

[b]Source provides no values for this parameter.

[c]Astellas data on file.

References

5. Akizawa T, Iwasaki M, Yamaguchi Y, Majikawa Y, Reusch M. Phase 3, Randomized, Double-Blind, Active-Comparator (Darbepoetin Alfa) Study of Oral Roxadustat in CKD Patients with Anemia on Hemodialysis in Japan. J Am Soc Nephrol. 2020;31(7):1628-39.

6. Akizawa T, Iwasaki M, Otsuka T, Yamaguchi Y, Reusch M. Phase 3 Study of Roxadustat to Treat Anemia in Non-Dialysis-Dependant CKD. Kidney Int Rep. 2021;6(7):1810-28.

7. Akizawa T, Yamaguchi Y, Otsuka T, Reusch M. A Phase 3, Multicenter, Randomized, Two-Arm, Open-Label Study of Intermittent Oral Dosing of Roxadustat for the Treatment of Anemia in Japanese Erythropoiesis-Stimulating Agent-Naive Chronic Kidney Disease Patients Not on Dialysis. Nephron. 2020;144(8):372-82.

8. Akizawa T, Otsuka T, Reusch M, Ueno M. Intermittent Oral Dosing of Roxadustat in Peritoneal Dialysis Chronic Kidney Disease Patients with Anemia: A Randomized, Phase 3, Multicenter, Open-Label Study. Ther Apher Dial. 2020;24(2):115-25.

9. Akizawa T, Ueno M, Shiga T, Reusch M. Oral roxadustat three times weekly in ESA-naive and ESA-converted patients with anemia of chronic kidney disease on hemodialysis: Results from two phase 3 studies. Ther Apher Dial. 2020;24(6):628-41.

10. Chen N, Hao C, Liu BC, Lin H, Wang C, Xing C, et al. Roxadustat Treatment for Anemia in Patients Undergoing Long-Term Dialysis. N Engl J Med. 2019;381(11):1011-22.

11. Chen N, Hao C, Peng X, Lin H, Yin A, Hao L, et al. Roxadustat for Anemia in Patients with Kidney Disease Not Receiving Dialysis. N Engl J Med. 2019;381(11):1001-10.

12. Shutov E, Sulowicz W, Esposito C, Tataradze A, Andric B, Reusch M, et al. Roxadustat for the treatment of anemia in chronic kidney disease patients not on dialysis: a Phase 3, randomized, double-blind, placebo-controlled study (ALPS). Nephrol Dial Transplant. 2021;36(9):1629-39.

13. Fishbane S, El-Shahawy MA, Pecoits-Filho R, Van BP, Houser MT, Frison L, et al. Roxadustat for Treating Anemia in Patients with CKD Not on Dialysis: Results from a Randomized Phase 3 Study. J Am Soc Nephrol. 2021;32(3):737-55.

14. Coyne DW, Roger SD, Shin SK, Kim SG, Cadena AA, Moustafa MA, et al. Roxadustat for CKD-related Anemia in Non-dialysis Patients. Kidney Int Rep. 2021;6(3):624-35.

15. Barratt J, Andric B, Tataradze A, Schomig M, Reusch M, Valluri U, et al. Roxadustat for the treatment of anaemia in chronic kidney disease patients not on dialysis: a Phase 3, randomized, open-label, active-controlled study (DOLOMITES). Nephrol Dial Transplant. 2021;36(9):1616-28.

16. Provenzano R, Shutov E, Eremeeva L, Korneyeva S, Poole L, Saha G, et al. Roxadustat for anemia in patients with end-stage renal disease incident to dialysis. Nephrol Dial Transplant. 2021;36(9):1717-30.

17. Fishbane S, Pollock CA, El-Shahawy M, Escudero ET, Rastogi A, Van BP, et al. Roxadustat Versus Epoetin Alfa for Treating Anemia in Patients with Chronic Kidney Disease on Dialysis: Results from the Randomized Phase 3 ROCKIES Study. J Am Soc Nephrol. 2022;33(4):850-66.

18. Csiky B, Schomig M, Esposito C, Barratt J, Reusch M, Valluri U, et al. Roxadustat for the Maintenance Treatment of Anemia in Patients with End-Stage Kidney Disease on Stable Dialysis: A European Phase 3, Randomized, Open-Label, Active-Controlled Study (PYRENEES). Adv Ther. 2021;38(10):5361-80.

19. Charytan C, Manllo-Karim R, Martin ER, Steer D, Bernardo M, Dua SL, et al. A Randomized Trial of Roxadustat in Anemia of Kidney Failure: SIERRAS Study. Kidney Int Rep. 2021;6(7):1829-39.

20. Barratt J, Sulowicz W, Schomig M, Esposito C, Reusch M, Young J, et al. Efficacy and Cardiovascular Safety of Roxadustat in Dialysis-Dependent Chronic Kidney Disease: Pooled Analysis of Four Phase 3 Studies. Adv Ther. 2021;38(10):5345-60.
